# Association Between Three Lung Donor Scores and Lung Acceptance in DBD and DCD Donors

**DOI:** 10.3389/ti.2026.15901

**Published:** 2026-01-16

**Authors:** Fabian Iten, Julius Weiss, Simon Schwab, Franziska Beyeler, Thorsten Krueger, Angela Koutsokera, Macé Schuurmans, Isabelle Opitz, György Lang, Franz Immer

**Affiliations:** 1 Swisstransplant, Swiss National Foundation of Organ Donation and Transplantation, Bern, Switzerland; 2 Division of Thoracic Surgery, Lausanne University Hospital, Lausanne, Switzerland; 3 Division of Pneumology, Lausanne University Hospital, Lausanne, Switzerland; 4 Division of Pulmonology, University Hospital Zurich, Zurich, Switzerland; 5 Department of Thoracic Surgery, University Hospital Zurich, Zurich, Switzerland

**Keywords:** donation after circulatory death (DCD), donor lung acceptance, extended criteria donor, lung donor score, lung transplantation

## Abstract

The use of extended criteria donors (ECD) has become increasingly important in lung transplantation to address organ donor shortages. To better assess lung graft quality and optimize donor selection, several scores have been developed. This study assesses whether Swiss lung acceptance practice is associated with three validated lung donor scores — the Oto Score, Eurotransplant Score (ET), and Zurich Donor Score (ZDS) — in both DBD and DCD donors. Due to limited clinical data, certain parameters of the Oto and ET Scores were adapted (aOto and aET). Data from 1515 actual deceased donors between 01.07.2014 and 30.06.2024 were analyzed. Logistic regression and AUC-ROC analysis were used to evaluate the scores' discriminative ability. Results showed that all three scores were associated with lung acceptance, with AUC values indicating acceptable to moderate discriminative ability — 0.75 for aOto, 0.70 for aET, and 0.77 for ZDS — and DCD donors being consistently less likely to be accepted for lung transplantation compared to DBD donors. Nonetheless, all three scores showed limitations as standalone models. Developing a novel, nationally applicable Swiss prediction tool integrating current lung acceptance criteria and recipient factors could improve donor–recipient matching, support more efficient organ utilization, and potentially increase transplant activity.

## Graphical Abstract

**Figure:**
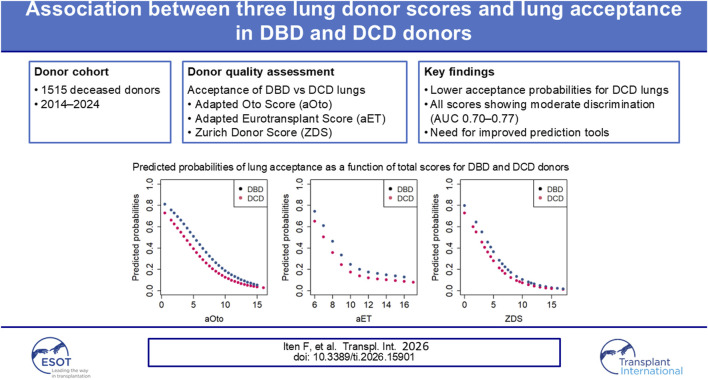

## Introduction

Lung transplantation is a well-established treatment for patients with end-stage lung diseases when all other therapeutic options are depleted. It significantly improves survival and quality of life [1]. These developments are further supported by recent large European series, including the Belgian national experience, demonstrating continued improvements in outcomes and expanding clinical applicability of lung transplantation [2]. However, the scarcity of transplantable donor lungs and the continually expanding waiting lists — as a consequence of broader indications and enhanced bridging options — in most countries remain substantial challenges in lung transplantation [3, 4]. To address this, the use of extended criteria donors (ECD) has increased in recent years, along with a growing use of donation after circulatory death (DCD) [5, 6]. Following a hiatus of 4-years, DCD organ donation was restarted in Switzerland in 2011, thereby enhancing transplant activity and reducing waiting times. By 2023, 41% of transplanted lungs in Switzerland were from DCD donors [7]. However, DCD donors differ in several ways from donation after brain death (DBD) donors. They are typically older, predominantly male, and more likely to have preexisting cardiac comorbidities. They also tend to have higher body mass index (BMI) and lower arterial partial pressure of oxygen/fraction of inspired oxygen (PaO_2_/FiO_2_) ratios, along with increased prevalence of preprocurement pneumonia [8, 9].

Despite the increasing use of ECD for lung transplantation, an international consensus on their uniform definition is lacking. In contrast, to provide a reference for optimal donor lung quality the International Society for Heart and Lung Transplantation (ISHLT) proposed five criteria in 2003 that define an ideal lung donor: age <55 years, smoking history <20 pack years, clear chest X-ray, no purulent secretions on bronchoscopy and a PaO_2_/FiO_2_ ratio >300 mmHg [10]. Building on these criteria, Oto and colleagues developed a score to numerically assess donor lung quality and predict the probability of organ acceptance [11]. Based on this score, but with certain modifications Smits and colleagues established the Eurotransplant Score (ET) which reliably predicted lung acceptance and 1-year survival [12]. To improve long-term outcome prediction, Ehrsam and colleagues proposed the Zurich Donor Score (ZDS) in 2020, incorporating diabetes and significant pulmonary infection as parameters, replacing bronchoscopy and chest X-ray findings [13].

This study aims to assess how the ZDS and adapted versions of the Oto and ET Scores are associated with lung acceptance practices in Switzerland. Given the increasing number of DCD donations in recent years, it is of particular interest to calculate scores separately for DBD and DCD to evaluate whether they exhibit different probabilities for transplantation.

## Patients and Methods

### Donor Data Collection

In this retrospective cohort study, we analyzed data from all deceased organ donors in Switzerland between 01.07.2014 and 30.06.2024 (n = 1730). Donor data were extracted from the Swiss Organ Allocation System (SOAS). SOAS is primarily designed for donor evaluation and organ allocation and does not systematically capture post-transplant recipient outcome data. Such data were not available for analysis and could not be included in the present study. For this analysis, only actual deceased donors (ADD) were included, whereas not utilized donors (n = 203) and potential donors with a positive SARS-CoV-2 PCR test during the period when SARS-CoV-2 was considered a contraindication for lung transplantation in Switzerland (between 12.03.2020 and 31.03.2023, n = 12) were excluded.

ADD refers to a deceased donor from whom at least one organ was procured for transplantation [14], whereas a not utilized donor is a deceased donor from whom no organs were recovered for transplantation. We categorized lung offers as either “refused” or “accepted,” to indicate that it is the transplant center decision whether to accept or not an offered lung graft for transplantation. The category “accepted lung offer” is equivalent to the outcome “lung transplanted,” while “refused lung offer” is equivalent to the outcome “lung not transplanted.” This decision can be taken based on the medical information during allocation or onsite during the procurement due to intraoperative findings. Additionally, for donor lungs in which no compatible recipient was on the waiting list are also in the category “refused lung offer.” In this study, accepted lung offers from Swiss donors also included lungs transplanted abroad. This occurred under international agreements when no suitable recipient was found on the national waiting list [15].

**FIGURE 1:**
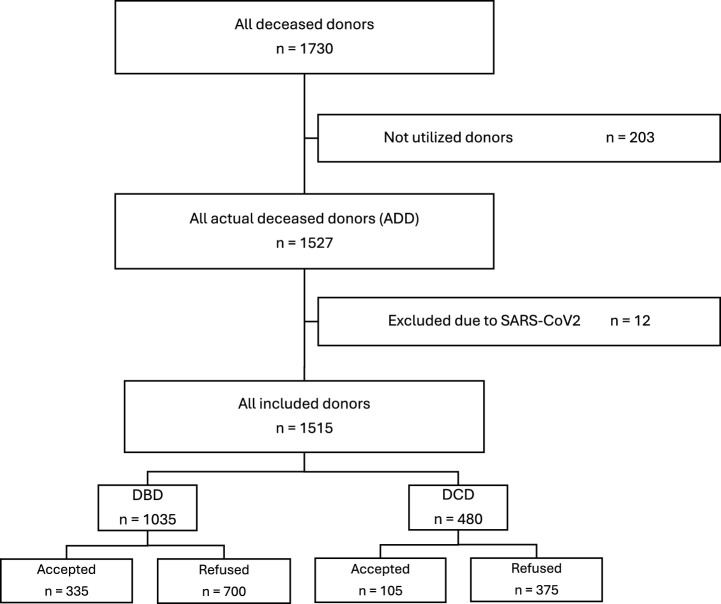
Flow chart of all reported deceased donors in Switzerland in 2014–2024.

An overview of the analyzed cases, detailing donor types and exclusion criteria, is presented in Figure 1.

### Adapted Oto Score (aOto)

Based on the five parameters defined by the ISHLT as ideal lung donor criteria, Oto and colleagues established their score to assess donor lung quality [11]. Lower score values were associated with better organ quality and, subsequently, higher lung acceptance. Since some clinical data relevant to the score calculation are not routinely recorded in the SOAS, we adapted the following parameters for this analysis:

- *Chest X-ray findings*: Due to the lack of qualitative assessment and opacity localization in the SOAS, this parameter was modified using analogous distribution of points as in the adapted ET Score (see below): Clear chest X-ray, atelectasis or edema was weighted 0.5 points; opacity or consolidation was weighted 2.5 points.
- *Secretions in bronchoscopy*: As the amount of bronchial secretions is not recorded in the SOAS, this parameter was simplified into two categories: “no secretions” was assigned 0.5 points, while “secretions” was assigned 2.5 points.
- Parameters with missing data were assigned score values following the methodology used in the ET Score [12].

Resulting from these adaptations, the maximum achievable score was 17, rather than 18 as in the original Oto Score [11].

### Adapted Eurotransplant Score (aET)

In the retrospective study by Smits and colleagues, the Eurotransplant Score was defined and empirically validated, building on the Oto Score and adding a parameter for donor history factors [12]. Parameters were weighted based on the odds ratios for lung acceptance or discard, with lower score values indicating better organ quality and, consequently, higher lung acceptance. For our analysis, we adjusted the following parameter:

- Donor history: In the original score, donor history was considered “compromised” in cases of drug abuse, malignancy, sepsis, meningitis or a positive virology status (HBsAg, HBcAb, HCVAb, anti-CMV) [12]. As the criterion “sepsis” is not routinely recorded in the SOAS and HBsAg as well as anti-CMV were rarely measured in the donor cohort of this period, donor history was considered “compromised” only in cases of drug abuse, malignancy, meningitis or positive virology status (HBcAb and/or HCVAb).

### Zurich Donor Score (ZDS)

Ehrsam and colleagues proposed and validated the ZDS on a Swiss donor and recipient cohort between 1992 and 2015 [13], identifying score parameters through univariate Cox regression and international consensus criteria, using more recent studies to determine point distribution compared to the Oto and ET Scores. Parameters with missing data were assigned score values following the methodology used in the ET Score [12]. Similar in the other scores, a lower ZDS indicates better organ quality and improved recipient survival.

### Statistical Analysis

The study population was subdivided into four groups: DBD and DCD donors, as well as accepted and refused lung offers. Donor characteristics of the groups were compared using Pearson’s Chi-squared test for categorical variables and Wilcoxon rank-sum test for continuous variables. Total aOto, aET and ZDS values were compared between accepted and refused DBD and DCD lung offers using Wilcoxon rank-sum test with p-values adjusted for multiple testing using the Bonferroni correction. Unless otherwise stated, the statistical analyses were performed using a two-sided approach and p-values of <0.05 were considered statistically significant. Multiple logistic regression was used to calculate the odds ratios (OR) for DCD donors and score values in relation to the chance of lung acceptance. To evaluate the discriminative ability of the aET, aOto, and ZDS in determining whether a lung donation is accepted or refused, the area under the receiver operating characteristic curve (AUC-ROC) was calculated. An AUC value of 0.5 signifies discriminative power no better than random guessing, while a value approaching 1 reflects perfect performance in outcome differentiation.

All statistical analyses in this study were performed using R version 4.4.2 [16].

### Ethics Approval

This analysis of SOAS data does not fall under the Swiss Human Research Act; legal regulation in Switzerland distinguishes between research (which is subject to approval) and quality assurance (not subject to approval). The Ethics Committee of the Canton of Bern determined the study to be of the quality assurance type and therefore exempt from review and the requirement to obtain informed consent (BASEC-ID: Req-2025-00515).

## Results

### Donor Characteristics

Of the 1515 included donors, 1035 (68.3%) were DBD donors and 480 (31.7%) were DCD donors. Among DBD donors, 335 lungs (32.4%) were accepted for transplantation, while 700 (67.6%) were refused. In comparison, only 105 lungs (21.9%) from DCD donors were accepted, and 375 (78.1%) were refused. Overall, 408 lungs (26.9%) were transplanted in Switzerland and 32 (2.1%) abroad, resulting in a total of 440 transplants (29.0%), while the remaining 1075 lungs (71.0%) were refused. An overview of DBD and DCD donor characteristics is presented in Table 1.

**TABLE 1:** Baseline Characteristics of accepted and refused DBD and DCD lung offers.

Characteristics	Accepted lung offer			Refused lung offer		
	DBD n = 335^a^	DCD n = 105^a^	p-value^2^	DBD n = 700^a^	DCD n = 375^a^	p-value^2^
Sex	​	​	<0.001	​	​	<0.001
Female	180 (53.7%)	35 (33.3%)	​	291 (41.6%)	106 (28.3%)	​
Male	155 (46.3%)	70 (66.6%)	​	409 (58.4%)	269 (71.8%)	​
Age (years)	51.0 (34.0, 60.0)	58.0 (48.0, 65.0)	<0.001	60.0 (49.0, 72.0)	62.0 (51.0, 71.0)	0.322
Height (cm)	170.0 (165.0, 180.0)	175.0 (165.0, 180.0)	0.111	172.0 (165.0, 180.0)	174.0 (166.0, 180.0)	0.049
Weight (kg)	70.0 (64.0, 80.0)	80.0 (70.0, 88.0)	<0.001	77.0 (66.0, 88.0)	80.0 (68.0, 90.0)	0.055
BMI (kg/m^b^)	24.2 (22.0, 26.9)	25.8 (23.7, 28.5)	<0.001	25.5 (23.1, 28.3)	26.0 (23.3, 29.0)	0.168
Blood group	​	​	0.101	​	​	0.082
A	142 (42.4%)	45 (42.9%)	​	330 (47.1%)	188 (50.1%)	​
AB	3 (0.9%)	3 (2.9%)	​	22 (3.1%)	15 (4.0%)	​
B	34 (10.1%)	4 (3.8%)	​	58 (8.3%)	43 (11.5%)	​
O	156 (46.6%)	53 (50.5%)	​	290 (41.4%)	129 (34.4%)	​
Cause of brain injury	​	​	<0.001	​	​	<0.001
ANX	67 (20.0%)	46 (43.8%)	​	209 (29.9%)	207 (55.2%)	​
CDE + CHE	182 (54.3%)	37 (35.2%)	​	357 (51.0%)	97 (25.9%)	​
CTR	82 (24.5%)	20 (19.0%)	​	125 (17.9%)	50 (13.3%)	​
OTH	4 (1.2%)	2 (1.9%)	​	9 (1.3%)	21 (5.6%)	​
Resuscitation	​	​	0.011	​	​	<0.001
Yes	97 (29.0%)	44 (41.9%)	​	262 (37.4%)	199 (53.1%)	​
No	235 (70.1%)	58 (55.2%)	​	428 (61.2%)	170 (45.3%)	​
Missing	3 (0.9%)	3 (2.9%)	​	10 (1.4%)	6 (1.6%)	​
Heart disease	​	​	0.001	​	​	0.625
Yes	33 (9.9%)	24 (22.9%)	​	211 (30.1%)	123 (32.8%)	​
No	294 (87.7%)	77 (73.3%)	​	473 (67.6%)	245 (65.3%)	​
Missing	8 (2.4%)	4 (3.8%)	​	16 (2.3%)	7 (1.9%)	​
Hypertension	​	​	<0.001	​	​	0.262
Yes	75 (22.4%)	44 (41.9%)	​	292 (41.7%)	157 (41.9%)	​
No	249 (74.3%)	61 (58.1%)	​	385 (55.0%)	212 (56.5%)	​
Missing	11 (3.3%)	0 (0%)	​	23 (3.3%)	6 (1.6%)	​
Diabetes	​	​	0.097	​	​	0.157
Yes	15 (4.5%)	10 (9.5%)	​	84 (12.0%)	43 (11.5%)	​
No	317 (94.6%)	95 (90.5%)	​	602 (86.0%)	330 (88.0%)	​
Missing	3 (0.9%)	0 (0%)	​	14 (2.0%)	2 (0.5%)	​

^a^ n (%); Median (Q1, Q3).

^b^ Pearson’s Chi-squared test; Wilcoxon rank sum test.

Abbreviations: ANX, anoxia; CDE, cerebral disease; CHE, cerebral hemorrhage; CTR, cerebral trauma; OTH, others.

DBD donors whose lungs were accepted were significantly younger, with a median age of 51.0 years (IQR: 34.0–60.0), compared to DCD lung donors whose median age was 58.0 years (IQR: 48.0–65.0; p < 0.001). Among donors whose lungs were refused, the age difference was less pronounced: DBD donors had a median age of 60.0 years (IQR: 49.0–72.0), and DCD donors 62.0 years (IQR: 51.0–71.0; p = 0.322).

Differences in causes of brain injury were found between DBD and DCD donors: The leading cause of death in DBD donors was cerebral disease or cerebral hemorrhage (54.3% among those whose lungs were accepted vs. 51.0% among those whose lungs were refused), whereas most DCD donors died from anoxic brain injury (43.8% vs. 55.2%, respectively). Preclinical resuscitation was significantly less common in DBD compared to DCD donors, both among those whose lungs were accepted (29.0% vs. 41.9%; p = 0.011) and those whose lungs were refused (37.4% vs. 53.1%; p < 0.001). Among donors whose lungs were accepted, DBD donors had lower rates of cardiovascular diseases (9.9% vs. 22.9%; p = 0.001), arterial hypertension (22.4% vs. 41.9%; p < 0.001), and diabetes (4.5% vs. 9.5%; p = 0.097) compared to DCD donors.

### Score Composition and Donor Distribution

Tables 2–4 present how the aOto, aET, and ZDS are built and how the different parameters are weighted with points. For each parameter, the respective donor distribution is shown.

**TABLE 2:** Formation of the aOto Score (aOto) with distribution of accepted and refused DBD and DCD lung offers.

Score parameters	Score points	DBD (n = 1035)		DCD (n = 480)	
		Accepted lung offer; n (%)	Refused lung offer; n (%)	Accepted lung offer; n (%)	Refused lung offer; n (%)
Total	​	335 (32.4%)	700 (67.6%)	105 (21.9%)	375 (78.1%)
Age (years)					
<45	0	125 (37.3%)	141 (20.1%)	19 (18.1%)	55 (14.7%)
45–54	1	82 (24.5%)	115 (16.4%)	20 (19.0%)	65 (17.3%)
55–59	2	37 (11.0%)	80 (11.4%)	22 (21.0%)	35 (9.3%)
>59	3	91 (27.2%)	364 (52.0%)	44 (41.9%)	220 (58.7%)
Smoking history (PY)					
<20	0	249 (74.3%)	330 (47.1%)	73 (69.5%)	177 (47.2%)
20–39	1	37 (11.0%)	114 (16.3%)	18 (17.1%)	47 (12.5%)
40–59	2	9 (2.7%)	94 (13.4%)	4 (3.8%)	58 (15.5%)
>59	3	6 (1.8%)	53 (7.6%)	1 (1.0%)	37 (9.9%)
Missing	0	34 (10.1%)	109 (15.6%)	9 (8.6%)	56 (14.9%)
Chest X-ray					
Clear, edema or atelectasis	0.5	213 (63.6%)	302 (43.1%)	54 (51.4%)	118 (31.5%)
Shadow or consolidation	2.5	122 (36.4%)	382 (54.6%)	51 (48.6%)	220 (58.7%)
Missing	0	0 (0.0%)	16 (2.3%)	0 (0.0%)	37 (9.9%)
Secretions in bronchoscopy					
No	0.5	11 (3.3%)	12 (1.7%)	4 (3.8%)	7 (1.9%)
Yes	2.5	49 (14.6%)	105 (15.0%)	17 (16.2%)	34 (9.1%)
Missing	0	275 (82.1%)	583 (83.3%)	84 (80.0%)	334 (89.1%)
PaO_2_/FiO_2_ (mmHg)					
>450	0	71 (21.2%)	41 (5.9%)	17 (16.2%)	31 (8.3%)
351–450	2	129 (38.5%)	140 (20.0%)	39 (37.1%)	76 (20.3%)
301–350	4	63 (18.8%)	103 (14.7%)	16 (15.2%)	64 (17.1%)
<301	6	72 (21.5%)	411 (58.7%)	33 (31.4%)	190 (50.7%)
Missing	3	0 (0.0%)	5 (0.7%)	0 (0.0%)	14 (3.7%)

**TABLE 3:** Formation of the aET Score (aET) with distribution of accepted and refused DBD and DCD lung offers.

Score parameters	Score points	DBD (n = 1035)		DCD (n = 480)	
		Accepted lung offer; n (%)	Refused lung offer; n (%)	Accepted lung offer; n (%)	Refused lung offer; n (%)
Total	​	335 (32.4%)	700 (67.6%)	105 (21.9%)	375 (78.1%)
Age (years)					
<55	1	207 (61.8%)	256 (36.6%)	39 (37.1%)	120 (32.0%)
55–59	2	37 (11.0%)	80 (11.4%)	22 (21.0%)	35 (9.3%)
>59	3	91 (27.2%)	364 (52.0%)	44 (41.9%)	220 (58.7%)
Smoking history					
No	1	171 (51.0%)	240 (34.3%)	52 (49.5%)	126 (33.6%)
Yes	2	130 (38.8%)	351 (50.1%)	44 (41.9%)	193 (51.5%)
Missing	1	34 (10.1%)	109 (15.6%)	9 (8.6%)	56 (14.9%)
Chest X-ray					
Clear, edema or atelectasis	1	213 (63.6%)	302 (43.1%)	54 (51.4%)	118 (31.5%)
Shadow or consolidation	2	122 (36.4%)	382 (54.6%)	51 (48.6%)	220 (58.7%)
Missing	1	0 (0.0%)	16 (2.3%)	0 (0.0%)	37 (9.9%)
Secretions in bronchoscopy					
Clear or nonpurulent	1	27 (8.1%)	29 (4.1%)	4 (3.8%)	15 (4.0%)
Purulent	2	15 (4.5%)	56 (8.0%)	5 (4.8%)	8 (2.1%)
Inflammatory	3	18 (5.4%)	32 (4.6%)	12 (11.4%)	18 (4.8%)
Missing	1	275 (82.1%)	583 (83.3%)	84 (80.0%)	334 (89.1%)
PaO_2_/FiO_2_ (mmHg)					
>350	1	200 (59.7%)	181 (25.9%)	56 (53.3%)	107 (28.5%)
301–350	2	63 (18.8%)	103 (14.7%)	16 (15.2%)	64 (17.1%)
≤300	3	72 (21.5%)	411 (58.7%)	33 (31.4%)	190 (50.7%)
Missing	2	0 (0.0%)	5 (0.7%)	0 (0.0%)	14 (3.7%)
Donor history					
Compromised	4	58 (17.3%)	165 (23.6%)	26 (24.8%)	69 (18.4%)
Uncompromised	1	277 (82.7%)	532 (76.0%)	79 (75.2%)	305 (81.3%)
Missing	1	0 (0.0%)	3 (0.4%)	0 (0.0%)	1 (0.3%)

**TABLE 4:** Formation of the Zurich Donor Score (ZDS) with distribution of accepted and refused DBD and DCD lung offers.

Score parameters	Score points	DBD (n = 1035)		DCD (n = 480)	
		Accepted lung offer; n (%)	Refused lung offer; n (%)	Accepted lung offer; n (%)	Refused lung offer; n (%)
Total	​	335 (32.4%)	700 (67.6%)	105 (21.9%)	375 (78.1%)
Age (years)					
<50	0	159 (47.5%)	183 (26.1%)	30 (28.6%)	78 (20.8%)
50–69	2	145 (43.3%)	307 (43.9%)	62 (59.0%)	186 (49.6%)
>69	5	31 (9.3%)	210 (30.0%)	13 (12.4%)	111 (29.6%)
Smoking history (PY)					
<20	0	249 (74.3%)	330 (47.1%)	73 (69.5%)	177 (47.2%)
20–49	3	43 (12.8%)	172 (24.6%)	20 (19.0%)	87 (23.2%)
>49	4	9 (2.7%)	89 (12.7%)	3 (2.9%)	55 (14.7%)
Missing	0	34 (10.1%)	109 (15.6%)	9 (8.6%)	56 (14.9%)
Diabetes mellitus					
No	0	317 (94.6%)	602 (86.0%)	95 (90.5%)	330 (88.0%)
Yes	2	15 (4.5%)	84 (12.0%)	10 (9.5%)	43 (11.5%)
Missing	0	3 (0.8%)	14 (2.0%)	0 (0.0%)	2 (0.5%)
PaO_2_/FiO_2_ (mmHg)					
>300	0	263 (78.5%)	284 (40.6%)	72 (68.6%)	171 (45.6%)
151–300	2	63 (18.8%)	285 (40.7%)	30 (28.6%)	138 (36.8%)
≤150	3	9 (2.7%)	126 (18.0%)	3 (2.9%)	52 (13.9%)
Missing	1.5	0 (0.0%)	5 (0.7%)	0 (0.0%)	14 (3.7%)
Significant pulmonary infection					
No	0	158 (47.2%)	183 (26.1%)	29 (27.6%)	70 (18.7%)
Yes	3	177 (52.8%)	500 (71.4%)	76 (72.4%)	274 (73.1%)
Missing	0	0 (0.0%)	17 (2.4%)	0 (0.0%)	31 (8.3%)

### Association of Scores and Donor Type With Lung Acceptance

**FIGURE 2:**
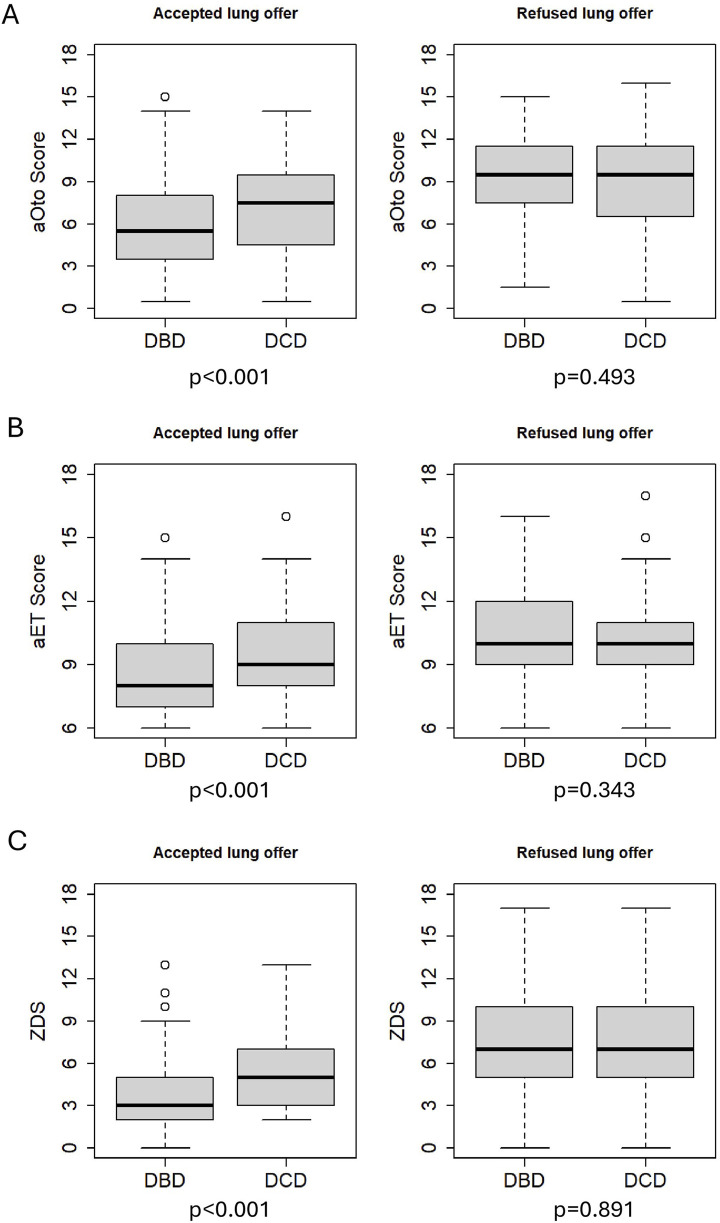
Comparison of total scores between accepted DBD and DCD as well as refused DBD and DCD lung offers. p-values (Bonferroni-adjusted for multiple testing) <0.001 for accepted lung offers. p = 0.493 for refused lung offers of aOto **(A)**, p = 0.343 for refused lung offers of aET **(B)** and p = 0.891 for refused lung offers of ZDS **(C)**.

The results of the regression analysis and odds ratios for lung acceptance are presented in Table 5. There is strong evidence that higher score values in aOto, aET and ZDS are associated with a decreased chance (OR <1) of donor lung acceptance in the analyzed Swiss study population (p < 0.001). For example, an increase in the ZDS from 5.0 to 8.5 reduced the odds of donor lung acceptance by a factor of 0.31 (95% CI 0.26–0.37). Similarly, aOto and aET showed odds ratios of 0.23 (95% CI 0.18–0.28) and 0.29 (95% CI 0.24–0.35), respectively. Across all three scores, DCD donors were significantly less likely to be accepted for lung transplantation than DBD donors, with odds ratios of 0.62 (95% CI 0.47–0.82) for aOto, 0.65 (95% CI 0.50–0.84) for aET and 0.68 (95% CI 0.52–0.90) for ZDS. Figure 2 shows that accepted lungs from DCD donors had significantly higher aOto (2-A), aET (2-B) and ZDS (2-C) compared to those of DBD donors (all p < 0.001, Bonferroni-adjusted for multiple testing). In contrast, no significant differences were observed between refused lungs from DBD and DCD donors (aOto: p = 0.493 (2-A); aET: p = 0.343 (2-B); ZDS: p = 0.891 (2-C)).

**TABLE 5:** Logistic regression model assessing the chance of lung acceptance based on donor type (DBD vs. DCD) and the corresponding score (aOto, aET, ZDS).

Scores	Interqartile difference	Odds ratio (95%-CI)	Chi-square	p-value^a^
aOto score				
Cadaveric donor type	–	–	11.5	<0.001
Cadaveric donor type DCD	–	0.62 (0.47–0.82)	–	–
aOto score	5.00 (5.50–10.50)	0.23 (0.18–0.28)	198	<0.001
Total	–	–	209	<0.001
aET score				
Cadaveric donor type	–	–	10.3	0.001
Cadaveric donor type DCD	–	0.65 (0.50–0.84)	–	–
aET score	3.00 (8.00–11.00)	0.29 (0.24–0.35)	151	<0.001
Total	–	–	164	<0.001
ZDS				
Cadaveric donor type	–	–	7.52	0.006
Cadaveric donor type DCD	–	0.68 (0.52–0.90)	–	–
ZDS	3.50 (5.00–8.50)	0.31 (0.26–0.37)	224	<0.001
Total	–	–	233	<0.001

Abbreviations: 95%-CI, 95% Confidence Interval.

^a^ Pearson’s Chi-squared test.

**FIGURE 3:**
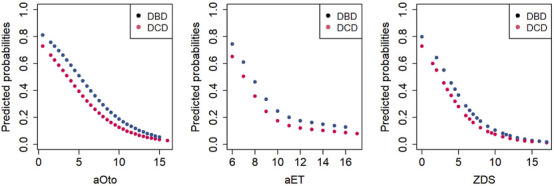
Predicted probabilities of lung acceptance as a function of total scores for DBD and DCD donors.

Predicted probabilities for each score value have been calculated separately for DBD and DCD donors based on the regression models, as shown in Figure 3. The predicted probability of a DBD lung donation being accepted was 79.8% at a ZDS of 0 (minimum score), whereas it was 1.6% at a ZDS of 19 (maximum score). Similarly, the predicted probability of a DCD lung donation being accepted was 72.8% at a ZDS of 0, whereas it was 1.1% at a ZDS of 19. Likewise, the aOto and aET returned high predicted probabilities of lung acceptance at low score values and correspondingly lower probabilities at higher values.

The AUC-ROC analysis assessing the discriminative ability of the three lung donor scores yielded a value of 0.75 (95% CI 0.72–0.78) for aOto, 0.70 (95% CI 0.67–0.73) for aET and 0.77 (95% CI 0.74–0.79) for ZDS, indicating an acceptable to moderate performance regarding the association with lung acceptance for DBD and DCD donors. Among the three lung donor scores, the ZDS demonstrated the strongest association with donor lung acceptance, as reflected by its highest discriminatory power.

## Discussion

### Comparison of DBD and DCD Donors

Our study demonstrated differences in donor characteristics between DBD and DCD donors whose lungs were accepted for transplantation. DCD donors were in median 7 years older and were more likely to be male, have higher BMI, and suffer more often from comorbidities such as heart disease, arterial hypertension and diabetes, compared to DBD donors. Furthermore, DCD donors whose lungs were accepted, more often had a smoking history of >20 pack years, pathological chest X-ray findings, significant pulmonary infections and thus lower PaO_2_/FiO_2_ ratios. Specifically, DCD donor characteristics such as older age, male predominance, higher BMI and lower PaO_2_/FiO_2_ ratios have also been described in studies from other countries [6, 9, 17].

Regarding the causes of brain injury, anoxic brain injury was more frequent among DCD donors, which is consistent with the results of earlier studies in the US and Spain [6, 9, 18]. DCD donors also underwent more often preclinical resuscitation, compared to DBD donors (50.6% of DCD donors vs. 34.7% of DBD donors). Furthermore, the resuscitation rate among Swiss organ donors increased from 24.1% (2007-2014) to 39.7% in our study population, possibly contributing to an increase in donors with irreversible hypoxic brain damage [19]. The higher resuscitation rate among DCD donors may increase the risk of aspiration, leading to a higher incidence of pulmonary infections and, consequently, lower PaO_2_/FiO_2_ ratios, as observed in the DCD donor characteristics.

### Association Between Scores and Lung Acceptance

All three scores showed acceptable to moderate discriminative ability in assessing lung acceptance with respect to AUC. An Austrian study reported similar results, with AUC values of 0.80 (95% CI 0.78–0.82) for the Oto and 0.71 (95% CI 0.68–0.73) for the ET Scores in a retrospective evaluation of 2201 donor lungs [20]. Likewise, a previous Swiss study analyzing 635 donors between 2007 and 2014 found a comparable AUC of 0.719 for their adapted ET Score [19]. This shows that our modifications to the ET and Oto Scores maintained comparable discriminatory power. Compared with the earlier Swiss studies by Elmer et al. [19] and Ehrsam et al. [13], our analysis is based on a more contemporary and comprehensive national donor cohort and evaluates all three established lung donor scores in parallel. Importantly, this study is also the first in Switzerland to separately analyze DBD and DCD donors, providing a more granular understanding of current lung acceptance practices across donor types. Among the analyzed lung donor scores, the ZDS showed the strongest association with the outcome. This may be due to regional differences in donor characteristics and organ quality assessment criteria, which could influence the applicability of different scores. Swiss transplant centers may prefer a nationally validated score over those developed on Australian or pan-European donor cohorts, like the Oto and ET Scores. Parameters such as diabetes and significant pulmonary infections, included in the ZDS but not in the aOto and aET, may therefore play a crucial role in assessing donor lung quality by Swiss transplant experts. However, it is not well established whether and to what extent the three lung donor scores are actually applied in clinical routine at the two Swiss transplant centers. This highlights the value of the present study, as it demonstrates that the scores align well with actual acceptance practices, even without confirmed clinical use.

### Impact of Donor Type on Scores

The regression model showed that DCD donors were less likely to be accepted for lung transplantation at each score value compared to DBD donors, likely because they met ISHLT’s ideal lung donor criteria less consistently, as reflected in the donor characteristics [10]. This is consistent with earlier studies reporting that DCD donors more often exhibit ECD characteristics [6]. However, a 2020 systematic review found no significant differences in 1-year survival or primary graft dysfunction between DBD and DCD lung recipients [21]. A 2025 meta-analysis even reported higher 5-year survival among DCD lung recipients in their study cohort, though differing donor characteristics may have influenced the result [22]. This discrepancy between the lower probability of being accepted for transplantation and comparable or superior recipient outcomes in international studies suggests potential for broader use of DCD lung donors [23]. It also questions the impact of ECD criteria on recipient outcomes. A recent European study found no significant difference in 1- and 5-year survival of recipients, regardless of whether the transplanted lungs came from deceased donors aged ≤30, 30-60, or ≥60 years [24]. Similar results were reported in a meta-analysis including several European and one American donor cohort [25]. Age – a key parameter in aET, aOto and ZDS – was significantly higher in DCD donors whose lungs were accepted, suggesting that, with careful and age-appropriate donor-recipient matching, more lungs from older DBD donors could potentially be utilized. Moreover, DCD donors whose lungs were accepted reached significantly higher score values than DBD donors (median 1–2 points higher; p < 0.001; see Figure 2). This may be an effect of the higher age of DCD donors which adds points to their score values.

### Limitations of the Scores

In our study, some DCD and DBD lungs were refused despite low scores, while others were accepted despite high scores. Such cases do not surprise, as factors that are not accounted for in the scores may influence lung quality and thus the chance of transplantation [26, 27]. Furthermore, in the publication by Smits and colleagues, the point distribution for the original ET Score involved statistically inappropriate handling of odds ratios, as they were added rather than multiplied [12]. Rounding of the derived score points may have further compromised the accuracy of the ET Score. Additionally, both the Oto and ET Scores are based on studies and consensus criteria from 1995 to 2008 [10, 11, 28], whereas advances in transplantation medicine have since led to improved outcomes for ECD lung grafts. For instance, a 2020 study showed that the ISHLT’s PaO_2_/FiO_2_ cutoff of 300 mmHg might be too restrictive, as recipients with ≤300 mmHg had similar short-term outcomes to those with >300 mmHg [29]. However, such findings highlight the value of lung donor scores, as they combine multiple donor criteria into a single tool to support more informed decisions on lung acceptance, rather than basing the decision on a single criterion.

Nevertheless, transplant decisions are influenced not only by donor characteristics but also by recipient and perioperative factors [30, 31] – none of which are accounted for in the Oto, ET and ZDS. A retrospective analysis from 2024 even showed that recipient characteristics might have a greater impact on post-transplant survival than donor characteristics [3].

Given these limitations, developing a novel, nationally applicable prediction tool for lung acceptance in Switzerland may be beneficial. A Swiss model could incorporate parameters that reflect up-to-date lung acceptance criteria as well as relevant recipient characteristics to enable more precise donor–recipient matching [3, 32].

### Limitations of the Study

There are some methodological limitations to our study. Adaptations were necessary to calculate the Oto and ET Scores, as certain parameters are not routinely recorded in the SOAS. Since bronchoscopy is optional before organ retrieval in Switzerland, this led to frequent missing data, potentially affecting aOto and aET results. Also, the interpretation of certain diagnostic tests, such as CT-scans and bronchoscopy, is somewhat subjective, which may also reduce score accuracy. Among the donors included in this study, the recorded reason for lung refusal may not always have been strictly donor-related, as it is often difficult to determine whether other factors – such as the lack of a compatible recipient – also influenced the transplant decision. Finally, this study confirmed that the three scores are associated solely with lung acceptance. To fully assess the suitability of the respective lung grafts, however, the scores would need to be validated based on short- and long-term recipient outcomes of the corresponding donor lungs.

Despite these limitations, this study offers novel insights into the assessment of lung acceptance practice in Switzerland. To the authors' knowledge, it is the first to separately analyze lung acceptance for DBD and DCD donors using the three scores.

### Conclusion

Our analysis demonstrated that the aOto, aET, and ZDS reliably reflected Swiss lung donor acceptance practice with acceptable to moderate discriminative ability. DCD donors were less likely to be accepted for lung transplantation at each score value, as they less frequently met ISHLT’s ideal lung donor criteria compared to DBD donors. However, recent studies suggest that outcomes of lung transplants from DCD donors are comparable to DBD donors, highlighting the potential for a broader use of DCD donors. Furthermore, the impact of *ex-vivo* lung perfusion in DCD donors has not been assessed in our study as this information was not available. Recent European Society for Organ Transplantation (ESOT) recommendations emphasize the importance of perfusion techniques, including *ex vivo* lung perfusion and normothermic regional perfusion, for donor lung assessment and optimization [33]. In addition, emerging evidence demonstrates that these technologies may expand donor suitability and improve graft evaluation, particularly in DCD donation pathways [34]. Future research should study the impact of *ex-vivo* perfusion on lung acceptance as well as on transplant outcomes.

Given the limitations of the scores, a novel prediction tool might be of practical use for lung transplantation in Switzerland. Such a tool should incorporate up-to-date lung acceptance criteria as well as relevant recipient characteristics. This could increase organ utilization, allowing more patients to benefit from transplantation. A prediction tool, however, should only serve as a complementary aid that cannot replace the decision-making of transplant experts. Further research on transplant outcomes from the donors in our cohort would be essential to fully evaluate the potential of DCD donors and to guide future allocation strategies.

## Data Availability

The data analyzed in this study is subject to the following licenses/restrictions: Access to Swiss Organ Allocation System (SOAS) data requires permission from the Federal Office of Public Health. The website https://www.gate.bag.admin.ch/artx/ui/home contains information on research data access to SOAS. Requests to access these datasets should be directed to https://www.gate.bag.admin.ch/artx/ui/home.
